# A review of the Japanese *Cryptochironomus* Kieffer, 1918 (Diptera, Chironomidae)

**DOI:** 10.3897/zookeys.771.24220

**Published:** 2018-07-05

**Authors:** Chuncai Yan, Ting Liu, Wei Cao, Guangjun Zhao, Wenbin Liu

**Affiliations:** 1 Tianjin Key Laboratory of Animal and Plant Resistance, Tianjin Normal University, Tianjin, 300387, PR China

**Keywords:** *Cryptochironomus*, Japan, key, new combination, synonymy, taxonomy

## Abstract

The genus *Cryptochironomus* Kieffer, 1918 from Japan is reviewed based on material composed of male adults. New combinations of three species are established and they are re-described based on male adult of: *C. misumaiprimus* (Sasa & Suzuki, 1998), **comb. n.**, *C. tokaraefeus* (Sasa & Suzuki, 1995), **comb. n.** and *C. tonewabeus* (Sasa & Tanaka, 2002), **comb. n.** Additional taxonomic notes are also provided: *C. albofasciatus* (Staeger, 1839), which is a senior synonym of *Parachironomus inafegeus* Sasa, Kitami & Suzuki, 2001 and *C. rostratus* (Kieffer, 1921) as the senior synonym of *Paracladopelma inaheia* Sasa, Kitami & Suzuki, 2001. A key to the known adult males of the genus from Japan is given.

## Introduction

*Cryptochironomus* is a diverse genus and widely distributed worldwide. Kieffer (1918) erected this genus based on Chironomus (Cryptochironomus) chlorolobus Kieffer, 1918 as type species. Subsequently, the genus in all its life stages was studied by a number of authors (Townes 1945; Roback 1957; Curry 1958; Shilova 1966; Beck and Beck 1969; Sæther 1977, 2009; Sasa and Kikuchi 1995; Sasa 1998; Zorina 2000; Makarchenko et al. 2005; Silva et al. 2010; Yan et al. 2016).

Eight *Cryptochironomus* species were reported from Japan by Sasa (1998), Sasa and Kikuchi (1995) and Sasa and Ogata (1999): *C. albofasciatus* (Staeger), *C. hentonensis* Hasegawa & Sasa, *C. javae* Kieffer, *C. sauteri* (Kieffer), *C. tamaichimori* Sasa, *C. tamayoroi* Sasa & Ichimori, *C. tokaracedeus* Sasa & Suzuki and *C. jokaprimus* Sasa & Ogata. *Cryptochironomus sauteri* (Kieffer) was transferred to *Parachironomus* Lenz by Sublette& Sublette (1973). After re-examining the material of *Cryptochironomus* Kieffer, *Parachironomus* Lenz, and *Paracladopelma* Harnisch from Sasa’s collection, and comparing the generic characters as defined by Cranston et al. (1989), *Parachironomus misumaiprima* Sasa & Suzuki, 1998, *Parachironomus tokaraefea* Sasa & Suzuki, 1995, and *Parachironomus tonewabea* Sasa & Tanaka, 2001 are assigned to the genus *Cryptochironomus* based on the following characters: inferior volsella with strong setae and completely covered by the superior volsella, lacking microtrichia; gonocoxite and gonostylus are fused; gonostylus short and wide. Moreover, *Parachironomus inafegeus* Sasa, Kitami & Suzuki is a junior synonym of *Cryptochironomus albofasciatus* (Staeger, 1839), and *Paracladopelma inaheia* Sasa, Kitami & Suzuki is a junior synonym of *Cryptochironomus rostratus* (Kieffer, 1921). Thus, eleven valid species of the genus *Cryptochironomus* are currently recorded in Japan: *C. albofasciatus*, *C. hentonensis*, *C. javae*, *C. jokaprimus*, *C. misumaiprimus*, *C. rostratus*, *C. tamaichimori*, *C. tamayoroi*, *C. tokaracedeus*, *C. tokaraefeus*, and *C. tonewabeus*.

In this paper, the genus *Cryptochironomus* is reviewed from Japan. Three new combinations and two synonyms are established and re-described. A key to the known adult males of the genus from Japan is presented.

## Materials and methods

A large amount of material composed of adult males belonging to the genus *Cryptochironomus* was examined based on slide-mounted following the procedures outlined by Sæther (1969). Morphology and terminology follow Sæther (1980). All of the examined specimens are deposited in the Department of Zoology, National Science Museum, Tokyo, Japan.

Abbreviations used in text as follows:

**AR** antennal ratio = length of ultimate fagellomere: combined lengths of fagellomeres one to penultimate;

**fe** femur;

**HR** hypopygium ratio = gonocoxite length: gonostylus length;

**HV** hypopygium value = body length: gonostylus length × 10;

**LR** leg ratio: tarsomere length: tibia length;

**LR_1_** tarsomere I length: tibia length;

**p1–3** Legs (1–fore, 2–mid, 3–hind);

**R** Radius; R_1_, Radius 1; R_4+5_, Radius four and five;

**Ta1–5** tarsomeres 1–5;

**Ti** tibia;

**VR** ratio of length of Cu: length of M.

## Taxonomy

### Cryptochironomus albofasciatus

Taxon classificationAnimaliaDipteraChironomidae

(Staeger, 1839)

Chironomus albofasciatus Staeger 1839: 566.
Chironomus (Cryptochironomus) albofasciatus Staeger. Goetghebuer 1937–1954: 38.
Cryptochironomus albofasciatus (Staeger). Reiss 1968: 195; Pinder 1978: 116; Sasa and Kawai 1987: 16; Sasa 1988: 56; 1989: 76; 1990: 31; 1991: 84; Sasa and Okazawa 1991: 53; Sasa, Kitami and Suzuki 1999: 7; 2001: 5; Makarchenko et al. 2005: 408; Yan et al. 2016: 486-487.
Parachironomus inafegeus Sasa, Kitami & Suzuki, 2001: 6 **(syn. n.**).

#### Material examined.

China. ♂ (No. 05227) Fujian Province, Longyan City, Shanghang county, 3.v.1993, light trap, X. Wang. The type specimen of *Parachironomus inafegeus* Sasa, Kitami & Suzuki. Holotype, ♂ (No. 398: 65), on the shore of lake Inawashiro, insect net, 8.vi.2000.

#### Diagnostic characters.

Thorax yellowish brown with white stripes; anal point parallel-sided, slender; anal tergite bands “V”-shaped, not fused in the middle; superior volsella crescent-like; inferior volsella tuberculate with two small protuberances, bearing two strong apical setae, free of microtrichia.

#### Distribution.

China (Fujian Province); Russian Far East; Japan; Europe.

#### Remarks.

The holotype specimen of *Parachironomus inafegeus* Sasa, Kitami & Suzuki, 2001 mainly agrees with the description of *Cryptochironomus albofasciatus* (Staeger, 1839) by Goetghebuer (1937–195: 38, fig. 116), especially the characters of anal point, superior and inferior volsella, and gonostylus. Consequently, it must be considered a junior synonym of *Cryptochironomus albofasciatus* (Staeger, 1839). See also the species illustration in Yan et al. (2016: 487, fig 1.).

### Cryptochironomus hentonensis

Taxon classificationAnimaliaDipteraChironomidae

Hasegawa & Sasa, 1987

Cryptochironomus hentonensis Hasegawa & Sasa, 1987: 290; Sasa and Kawai 1987: 63; Sasa and Kikuchi 1995: 99; Sasa 1998: 28; Makarchenko et al. 2005: 409.

#### Material examined.

Japan. The type specimen of *Cryptochironomus hentonensis* Hasegawa & Sasa. Holotype, ♂ (No. 64: 91), on the bank of Eel pond at Hentona, Okinawa, in the Ryukyu Islands, southern Japan. 2.xii.1982.

#### Diagnostic characters.

AR 3.03; frontal tubercles present; anal point long and tapering, lateral setae and microtrichia absent; superior volsella thumb-like, inferior volsella finger-shaped.

#### Distribution.

Japan; Russian Far East.

### Cryptochironomus javae

Taxon classificationAnimaliaDipteraChironomidae

Kieffer, 1924

Cryptochironomus javae Kieffer, 1924: 264; Sublette and Sublette 1973: 403; Sasa and Hasegawa 1983: 323; Sasa and Kawai 1987: 61.

#### Diagnostic characters.

According to Sasa and Hasegawa (1983: 323, Fig. 3K). Total length 4.84 mm, wing length 2.22 mm. AR 2.44, LR_1_ 1. 89, frontal tubercles present. Mid and hind with two tibial spurs. Anal point short and wide. Superior volsella pad-like, bearing two setae at apex; inferior volsella tuberculate, covered by superior volsella completely, bearing two setae at apex, free microtrichia. Gonostylus wide and short, with almost straight inner margin, rounded apically.

#### Distribution.

Japan; Indonesia.

### Cryptochironomus jokaprimus

Taxon classificationAnimaliaDipteraChironomidae

Sasa & Ogata, 1999

Cryptochironomus jokaprimus Sasa & Ogata, 1999: 86; Sasa and Suzuki 2001: 2.

#### Material examined.

Japan. Holotype, ♂ (No. 321: 37), at the side of a stream discharging water (the Kurobe Municipal Sewage Treatment Plant (Kurobe Joka Center)), in urban areas of Japan, 27.viii.1996, light trap.

#### Diagnostic characters.

Frontal tubercles prominent, roughly conical. Tergite IX rounded at the posterior margin. Anal point concave in its median portion, and parallel-sided to the apex, apically rounded. Superior volsella narrow at base and strongly expanded distally, bearing five strong setae, inferior volsella semicircular, gonostylus with small protrusion at apex.

#### Distribution.

Japan.

### Cryptochironomus misumaiprimus

Taxon classificationAnimaliaDipteraChironomidae

(Sasa & Suzuki, 1998) comb. n.

Figure 1

Paracladopelma misumaiprima Sasa & Suzuki, 1998: 18.

#### Material examined.

Japan. Holotype, ♂ (No. 348: 30), at Misumai, Hokkaido, 6. ix. 1997, sweep net.

#### Diagnostic characters.

AR 2.84. LR_1_ 1.48. Anal point linear and distinctly parallel-sided, slender. Anal tergite bands “wide Y”-shaped. Superior volsella broadly semicircular; inferior volsella finger-shaped, slender at base, swelling at apex, bearing two setae, free microtrichia. Gonostylus tapering to the apex.

#### Description.

**Male imago** (n = 1).

Total length 5.45 mm; wing length 2.60 mm; total length / wing length 2.10; wing length / length of profemur 2.08.

*Coloration*. Thorax yellow-white, with yellow-brown spots. Femora of front legs yellow-brown, tibia and tarsi dark brown; femora, tibiae and tarsi I of mid and hind legs yellow-brown, tarsi II–V dark brown. Abdomen yellow-brown, hypopygium dark brown.

*Head.* AR: 2.84. Ultimate flagellomere 1080 μm; frontal tubercles present but unclear. Temporal setae 24, including ten inner verticals, six outer verticals, and eight postorbitals. Clypeus with 16 setae; palpomere lengths (µm): 48; 63; 245; 208; 260. Palp segment 5^th^/3^rd^: 1.06.

*Thorax*. Antepronotals unclear; acrostichals nine; dorsocentrals 16; prealars six. Scutellum with 24 setae.

*Wing*. VR: 1.10. R with 22 setae. R_1_ with 21 setae. R_4+5_ with 37 setae. Brachiolum with three setae. Squama with nine setae.

*Legs*. Front tibia with three subapical setae, 185 μm and 188 μm, the other lost. Mid legs with two spurs, 30 mm and 33 mm; tibial comb with 52 teeth, 14 mm long. Spurs of hind tibia 30 mm and 33 mm long, tibial comb with 62 teeth, 15 mm long. The sensilla chaetica of tarsus I of mid leg and metapedes were not distinguishable. Lengths (in µm) and proportions of thoracic legs as in Table 1.

**Table 1.** Male adult of *Cryptochironomus misumaiprimus* (Sasa & Suzuki, 1998), comb. n. Length (µm) and proportions of legs.

	fe	ti	ta_1_	ta_2_	ta_3_	ta_4_	ta_5_	LR
p_1_	1250	1000	1475	710 0	630	540	230	1. 48
p_2_	1150	1025	700	310	250	140	100	0. 68
p_3_	1250	1375	920	460	370	220	120	0. 67

**Figure 1.**
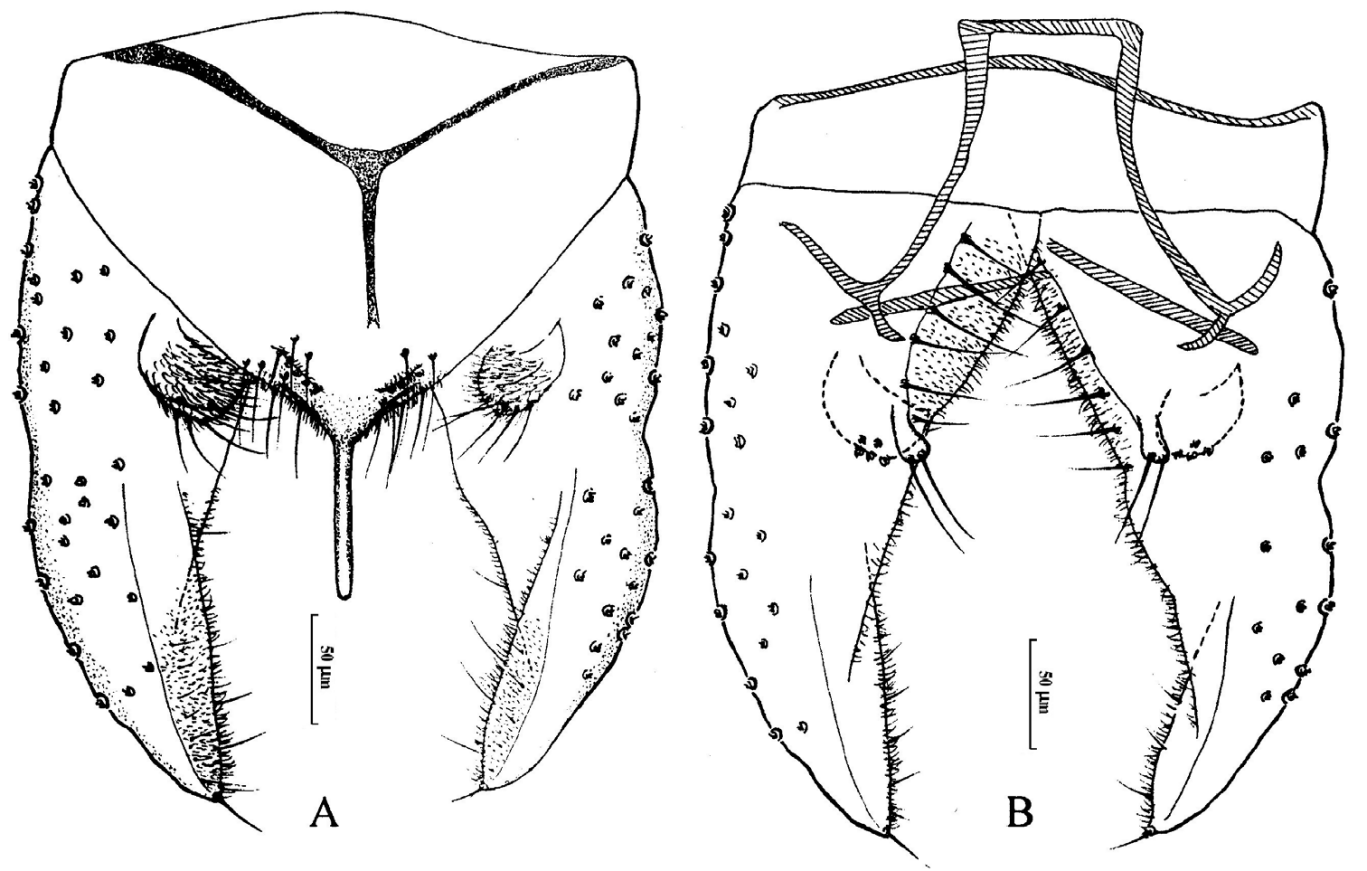
Male adult of *Cryptochironomus misumaiprimus* (Sasa & Suzuki, 1998), comb. n., male. **A** hypopygium (dorsal view) **B** hypopygium (ventral view).

*Hypopygium* (Fig. 1). Tergite IX with 11-13 setae placed dorsally and ventrally on each side of the base of anal point. Laterosternite IX with three lateral setae. Anal point parallel-sided, slender, lateral setae and microtrichia absent. Anal tergite bands “Y”-shaped. Phallapodeme 125 mm long. Transverse sternapodeme 80 mm long. Superior volsella semicircular, bearing five strong setae at apex, covered with microtrichia. Inferior volsella finger-shaped, swelling at apex, bearing two setae at apex, free microtrichia. Gonocoxite 140 mm long, bearing six strong setae along inner margin. Gonostylus 175 mm long, widest at base, curved slightly at 1/3 distance from base, tapered to the apex, bearing seven short setae along inner margin and one seta at apex. HR: 0.89; HV: 3.11.

#### Distribution.

Japan.

#### Remarks.

The characters of frontal tubercles, superior and inferior volsella, and gonostylus of this species followed the generic character of the genus *Cryptochironomus* emended by Cranston et al. (1989). The small frontal tubercles and “Y”-shaped anal tergite bands separate this species from other members of the genus.

### Cryptochironomus rostratus

Taxon classificationAnimaliaDipteraChironomidae

(Kieffer, 1921)

Figure 2

Chironomus rostratus Kieffer, 1921: 67.
Chironomus (Chironomus) rostratus : Edwards 1929: 390.
Chironomus (Cryptochironomus) rostratus : Goetghebuer 1928: 84; Kruseman 1933: 187.
Cryptochironomus rostratus : Pinder 1978: 116; Ree and Kim 1981: 143; Cranston and Judd 1989: 252; Chaudhuri and Chattopadhyay 1990: 157; Dutta et al. 1996: 269; Wang 2000: 643.
Paracladopelma inaheia Sasa, Kitami & Suzuki, 2001: 7 (**syn. n.**).

#### Material examined.

Japan: the type specimen of *Paracladopelma inaheia* Sasa, Kitami & Suzuki, Holotype, ♂ (No. 402: 17), shore of Lake Inawashiro, Japan, 21.viii.2000, light trap.

#### Diagnostic characters.

Thorax with dark brown spots; the posterior margin of tergite IX shoulder-like or slightly cone-like; anal point slender, tapering distally or parallel-sided. Superior volsella crescent-like; inferior volsella tuberculate, bearing 1-3 stout setae, free microtrichia. Anal tergite bands “V” shaped, the junction of gonostylus and gonocoxite distinctly concaved, curved at 1/3 distance from base, apex with a small protrusion, bent inwards and bearing one seta.

#### Description.

**Male imago (n = 1)**.

Total length 5.45 mm; wing length 2.30 mm, total length/wing length 2.37; wing length / length of profemur 2.17.

*Coloration*. Thorax yellow-white, with yellow-brown spots. Femora of front legs yellow-brown, tibiae and tarsomeres dark brown; femora and tibiae of mid and hind legs yellow-brown, tarsi I yellow-brown except for dark yellow-brown at ends, tarsi II–V dark yellow brown. Abdomen yellow-brown, hypopygium dark brown.

*Head*. AR: 2.89. Ultimate flagellomere 1010 μm; little and semi-circular frontal tubercles, diameter 7 μm. Temporal setae 25, including six inner verticals, 12 outer verticals, and seven postorbitals. Clypeus with 17 setae. Palpomere lengths (µm): 45; 65; 220; 212; 230. Palp segment 5^th^/3^rd^. 1.05.

*Thorax*. Antepronotals bare; acrostichals ten; dorsocentrals 14; prealars eight. Scutellum with 32 setae.

*Wing*. VR: 1.12. R with 24 microtrichia. R_1_ with 23. R_4+5_ with 22 setae. Brachiolum with three strong setae. Squama with 17 fringed setae.

*Legs*. Front tibia with three subapical setae, 165μm and 168μm, the other lost. Mid legs with two spurs, 30 mm and 35 mm, tibial comb with 44 teeth, 15 mm long. Spurs of hind tibia 35 mm and 37 mm long, tibial comb with 60 teeth, 15 mm long. Tarsus I of mid leg with nine sensilla chaetica, Tarsus I of metapedes leg with six sensilla chaetica. Lengths (in µm) and proportions of thoracic legs as in Table 2.

**Table 2.** Male adult of *Cryptochironomus rostratus* (Kieffer, 1921). Lengths (µm) and proportions of legs.

	fe	ti	ta_1_	ta_2_	ta_3_	ta^4^	ta_5_	LR
p_1_	1060	825	1500	650 0	540	440	200	1.82
p_2_	1020	910	630	300	220	120	100	0.69
p_3_	1110	1200	860	450	350	190	120	0.72

**Figure 2.**
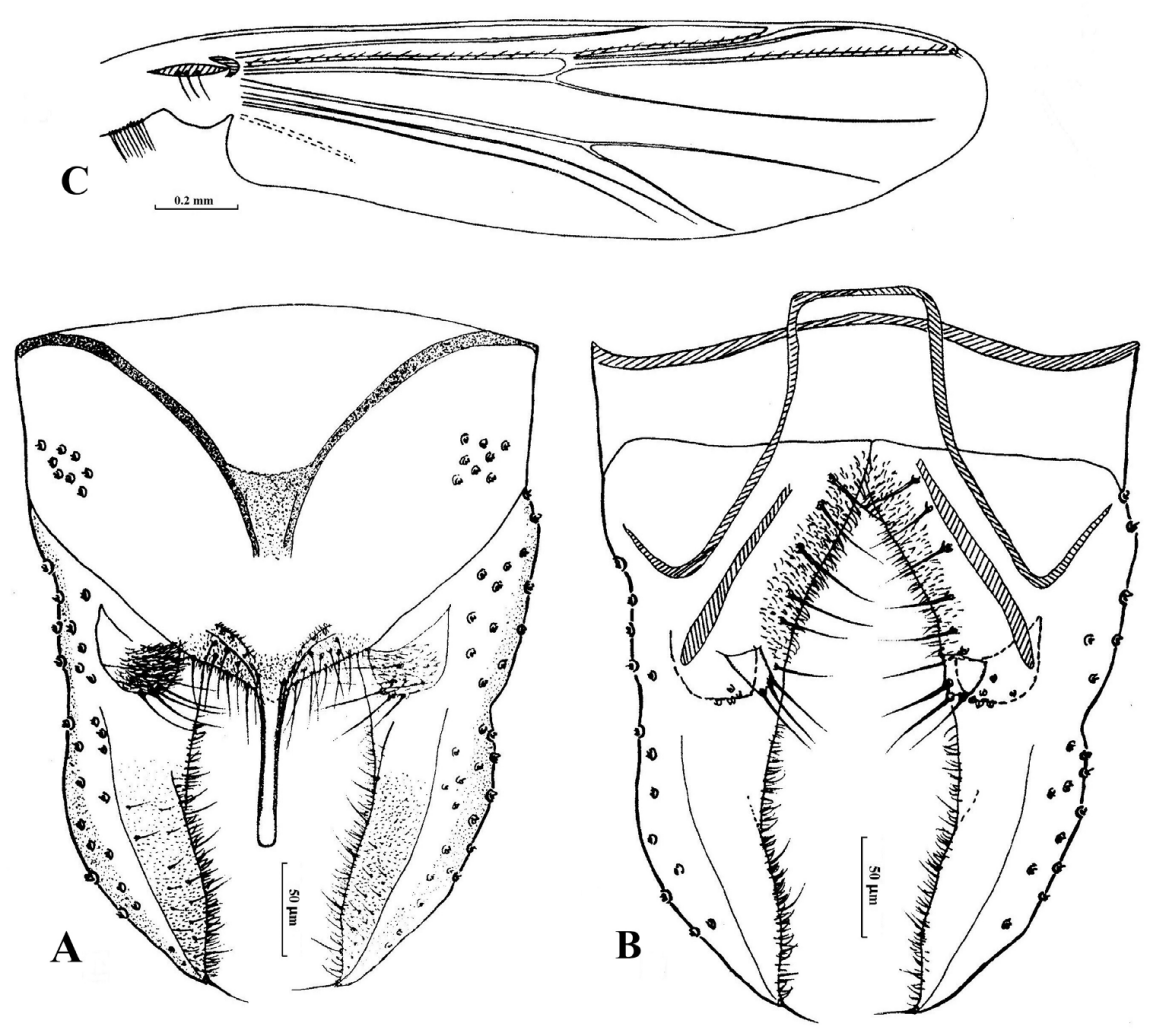
Male adult of *Cryptochironomus rostratus* (Kieffer, 1921) (holotype specimen of *Paracladopelma inaheia* Sasa, Kitami & Suzuki) male. **A**. hypopygium (dorsal view) **B**. hypopygium (ventral view) **C** Wing.

*Hypopygium* (Fig. 2). Tergite IX broad with cone-like posterior margin, bearing approximately 30 setae located dorsally and ventrally near the base of anal point. Laterosternite IX with six lateral setae. Anal point 88 mm long, slightly wider at base, almost parallel-sided, apically rounded, lateral setae and microtrichia absent. Anal tergite bands “V”-shaped. Phallapodeme 135 mm long. Transverse sternapodeme 75 mm long. Superior volsella spherical to bulb-like, covered with microtrichia, bearing six strong setae at apex. Inferior volsella tuberculate, bearing three setae at apex, free microtrichia. Gonocoxite 138 mm long, with six stout setae placed along inner margin. Gonostylus 170 mm long, base widest, slightly curved in the middle, tapered to the apex, bearing seven short setae along inner margin and one stronger seta at apex. HR: 0.81; HV: 3.21.

#### Distribution.

China (Zhejiang, Fujian, Jiangxi, Guangxi, Hainan, Sichuan, Guizhou, Tibet, Taiwan); South Korea; Japan; India; Bangladesh; Lebanon; Turkey; Europe (Germany, UK, Holland, Belgium).

#### Remarks.

The holotype of *Paracladopelma inaheia* belongs to the well-known European *Cryptochironomus rostratus* based on morphological characters of the hypopygium and metric measurements (Kieffer 1921; Pinder 1978: fig. 147B). Populations of *C. rostratus* from both Japan and other European areas key close together despite some minor morphological differences mainly related to geographical variation, especially the general shape of tergite IX, anal point, superior volsella, inferior volsella, and gonostylus. Thus, *Paracladopelma inaheia* can be considered as a junior synonym of *Cryptochironomus rostratus* (Kieffer, 1921).

### Cryptochironomus tamaichimori

Taxon classificationAnimaliaDipteraChironomidae

Sasa in Sasa & Kawai, 1987

Cryptochironomus sp. “*hentona*” Sasa & Ichimori, 1983: 103.
Cryptochironomus tamaichimori Sasa in Sasa & Kawai 1987: 61; Sasa 1993: 55; Makarchenko et al. 2005: 409.

#### Diagnostic characters.

Frontal tubercles and the posterior margin of tergite IX are highly variable in this species. The main distinguishing features are as follows: superior volsella bulbous to globular, both sides of the inferior volsella stretching upward at base, formed into flank-shaped, free microtrichia, bearing two long apical setae at apex. The junction of gonostylus and gonocoxite concaved obviously, gonostylus abruptly narrowed near apex and apically pointed, bearing one seta at apex.

#### Distribution.

China (Hebei, Zhejiang, Fujian, Hubei, Guangdong, Hainan, Sichuan); Japan; Russian Far East.

### Cryptochironomus tamayoroi

Taxon classificationAnimaliaDipteraChironomidae

Sasa & Ichimori, 1983

Cryptochironomus tamayoroi Sasa & Ichimori, 1983: 102; Sasa and Kawai 1987: 61

#### Material examined.

Japan. Holotype, ♂ (No. 74: 01), Tama River, at the Yoroi Bridge, at Yoroi-bashi, Japan. 11. iii. 1982.

#### Diagnostic characters.

AR 2.56. Frontal tubercles present; tergite IX bearing three setae, posterior margin tapering. Anal point parallel-sided. Anal tergite bands “V”-shaped. Superior volsella about twice as long as wide, with four long setae; inferior volsella extending well beyond posterior margin of superior volsella. Gonostylus wider at base and tapering towards apex, with one single apical seta.

#### Distribution.

Japan.

### Cryptochironomus tamayoroi

Taxon classificationAnimaliaDipteraChironomidae

Sasa & Suzuki, 1995

Cryptochironomus tokaracedeus Sasa & Suzuki, 1995: 260.

#### Material examined.

Japan. ♂ (No. 286: 21), edge of rice paddies, Tokara Islands, Kagoshima, Japan. 18–19. v. 1994, sweep net, Coll. H. Suzuki.

#### Diagnostic characters.

Frontal tubercles present. The tibia of front leg with one subapical seta. The posterior margin of tergite IX shoulder-like. Anal point tapering to the apex, lateral setae and microtrichia absent. Anal tergite bands “H”-shaped. Superior volsella small, thumb-like, almost entirely covered by microtrichia; inferior volsella bearing three apical setae at apex, free microtrichia.

#### Distribution.

Japan.

### Cryptochironomus tokaraefeus

Taxon classificationAnimaliaDipteraChironomidae

(Sasa & Suzuki, 1995) comb. n.

Figure 3

Paracladopelma tokaraefea Sasa & Suzuki, 1995: 262.

#### Material examined.

Japan. Holotype specimen of *Paracladopelma tokaraefea* Sasa & Suzuki, 1995. ♂ (No. 287: 19), at the edge of a rice paddy, on Kuchinoshima Island, on the Tokara Islands Kagoshima, Japan. 19.v.1994, insect net, Coll. H. Suzuki.

#### Diagnostic characters.

Anal point almost parallel-sided. Anal tergite bands H-shaped. Superior volsella bulbous to spherical; inferior volsella square-shaped, and width is equal to half of superior volsella, bearing three long setae at apex, free microtrichia.

#### Male imago

(n = 1). Total length 5.23 mm; wing length 2.20 mm; total length / wing length 2.38; wing length / length of profemur 2.26.

*Coloration*. Thorax yellow-white, with yellow-brown spots; femora, tibiae and tarsi I of mid and hind legs yellow-brown; tarsi II-V dark yellow-brown. Abdomen yellow brown; hypopygium dark brown.

*Head*. Antenna damaged; frontal tubercles unrecognizable. Temporal area damaged. Clypeus with 15 setae. Palpomere lengths (µm): 48; 52; 200; 180; 228. Palp segment 5^th^/3^rd^: 1.14.

*Thorax*. Antepronotals bare; acrostichals eight; dorsocentrals 13; prealars five. Scutellum with 28 setae.

*Wing*. VR: 1.08. R with 24 microtrichia. R_1_ with 19. R_4+5_ with 28 setae. Brachiolum with three strong setae. Squama with at least ten fringed setae.

*Legs*. Front tibia with two subapical setae, 150 μm and 155 μm. Mid legs with two spurs, 13 mm and 22 mm, tibial comb with 50 teeth, 10 mm long. Spurs of hind tibia 14 mm and 25 mm long, tibial comb with 66 teeth, 10 mm long. Tarsus I of mid and hind leg were not distinguishable. Lengths (in µm) and proportions of thoracic legs as in Table 3.

**Table 3.** Male adult of *Cryptochironomus tokaraefeus* (Sasa & Suzuki, 1995), comb. n. Length (µm) and proportions of legs.

	fe	ti	ta_1_	ta_2_	ta_3_	ta_4_	ta_5_	LR
p_1_	975	800	1325	550 0	500	400	180	1.66
p_2_	960	890	560	270	200	120	100	0.63
p_3_	1050	1115	810	400	300	270	120	0.73

**Figure 3.**
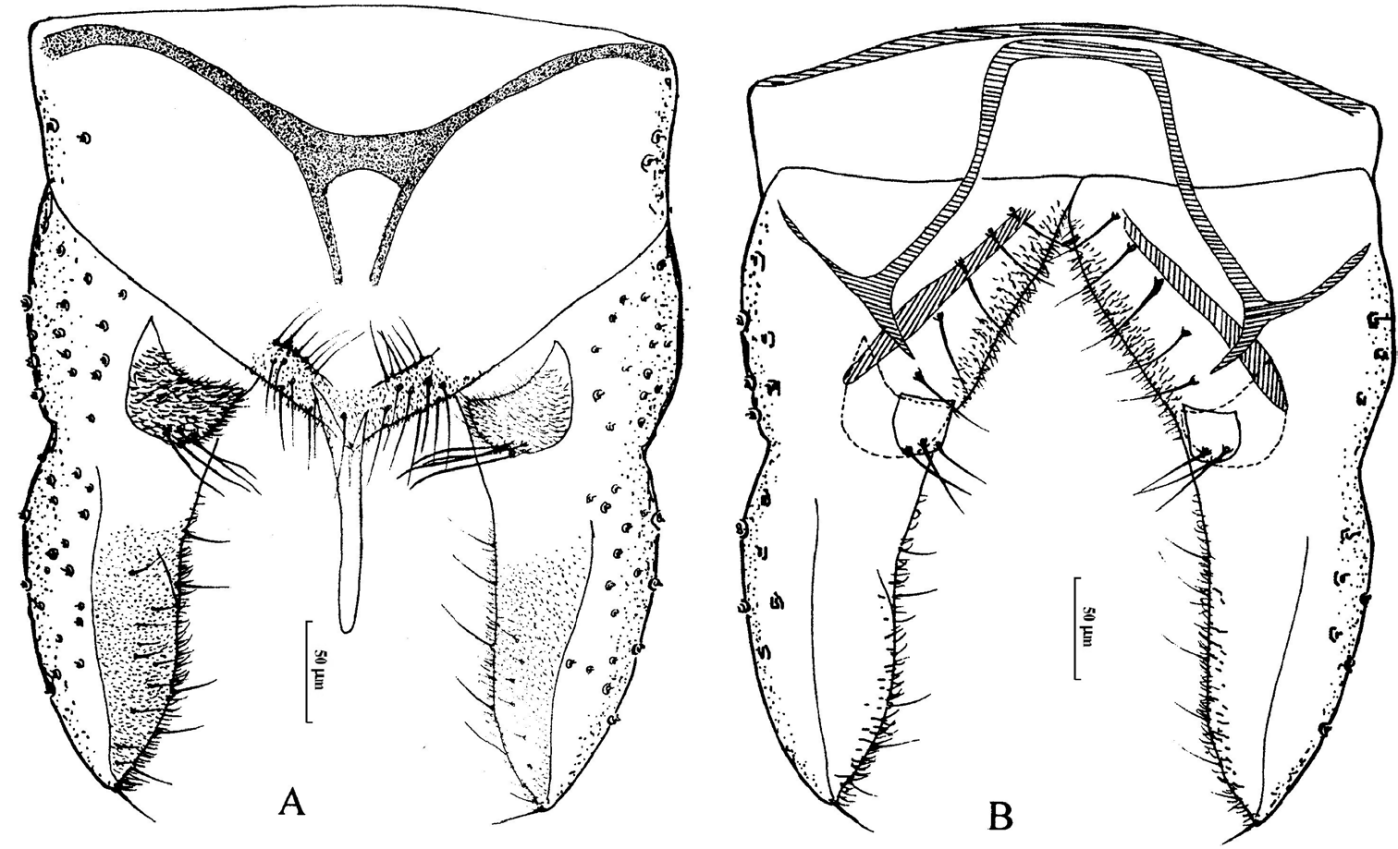
Male adult of *Cryptochironomus tokaraefeus* (Sasa & Suzuki, 1995), comb. n., male. **A** hypopygium (dorsal view) **B** hypopygium (ventral view)

*Hypopygium* (Fig. 3). Tergite IX bearing 14 setae. Laterosternite IX with two lateral setae. Anal point 100 mm long, straight, wider base, almost parallel-sided, narrower at apex, lateral setae and microtrichia absent. Anal tergite bands H-shaped. Phallapodeme 120 mm long. Transverse sternapodeme 85 mm long. Superior volsella semicircular, covered with microtrichia, bearing four strong setae along inner margin. Inferior volsella square-shaped, width and length are almost equal, bearing two long setae at apex, free microtrichia. Gonocoxite 138 mm long, bearing five strong setae along inner margin. Gonostylus 170 mm long, widest at basal 1/3, tapering to the apex. HR: 0.81; HV: 3.08.

#### Distribution.

Japan.

#### Remarks.

The characters of frontal tubercles, superior volsella and inferior volsella, and gonostylus followed the generic character of the genus *Cryptochironomus* by Cranston et al. (1989). The character of “H”-shaped anal tergite is similar to *Cryptochironomus tokaracedeus* Sasa & Suzuki, 1995, but *tokaraefeus* can be separated by the semicircular superior volsella, square-shaped inferior volsella and some metric characteristics.

### Cryptochironomus tonewabeus

Taxon classificationAnimaliaDipteraChironomidae

(Sasa & Tanaka, 2002) comb. n.

Figure 4

Paracladopelma tonewabea Sasa & Tanaka, 2002: 30.

#### Material examined.

Japan. Holotype specimen of *Paracladopelma tonewabea* Sasa & Tanaka, 2002. ♂ (No. 405: 51), Tone River at Taisho Bridge, Gunma Prefecture, Japan. 19. xii. 2000, reared from bottom sample, coll. N. Tanaka.

#### Diagnostic characters.

AR 2.53, frontal tubercles cylindrical. Tergite IX broadly semi-circular, with 16 setae (eight on each side of base of anal point). Anal point wide at base, parallel-sided medially and distally, rounded apically. Anal tergite bands “wide V”-shaped, not fused in the middle, and abruptly interrupted at the end. Superior volsella peanut-like, concave midially. Inferior volsella thumb-like, with slender extension at the base. Gonostylus and gonocoxite fused completely. Gonostylus wider at base, tapering to the apex.

*Coloration*. Thorax light yellow-brown, with yellow-brown spots, Femora of front legs yellow-brown, tibia and tarsomeres dark brown; femora, tibiae and tarsi I of mid and hind legs brown; tarsi II-V dark yellow-brown, hypopygium dark brown.

*Head*. AR: 2.53, Ultimate flagellomere 1010 μm; frontal tubercles tapering, 20 μm high, 13 μm width at base. Temporal setae 23, including seven inner verticals, nine outer verticals, and seven postorbitals. Clypeus with 20 setae. Palpomere lengths (µm): 60; 75; 290; 120; 330. Palp segment 5^th^/3^rd^: 1.14.

*Thorax*. Antepronotals bare; acrostichals ten; dorsocentrals ten; prealars six. Scutellum with 25 setae.

*Wing*. VR: 1.15. R with 22 microtrichia. R_1_ with 19. R_4+5_ with 28 setae. Brachiolum and squama were broken.

*Legs*. Front tibia with three subapical setae, 175 μm, 180 μm, 193 μm. Mid legs with three spurs, 28 μm, 30 μm; tibial comb with 48 teeth, 12 μm long. Spurs of hind tibia 30 μm and 37 μm long, tibial comb with 64 teeth, 13 μm long. Tarsus I of mid leg with three sensilla chaetica; tarsus I of metapedes leg with two sensilla chaetica. Lengths (in µm) and proportions of thoracic legs as in Table 4.

**Table 4.** Male adult of *Cryptochironomus tonewabeus* (Sasa & Tanaka, 2002), comb. n. Length (µm) and proportions of legs.

	fe	ti	ta_1_	ta_2_	ta_3_	ta_4_	ta_5_	LR
p_1_	1300	950	1700	780 0	650	600	225	1. 79
p_2_	1125	1030	690	320	240	140	100	0. 67
p_3_	1275	1330	920	450	380	210	110	0. 69

**Figure 4.**
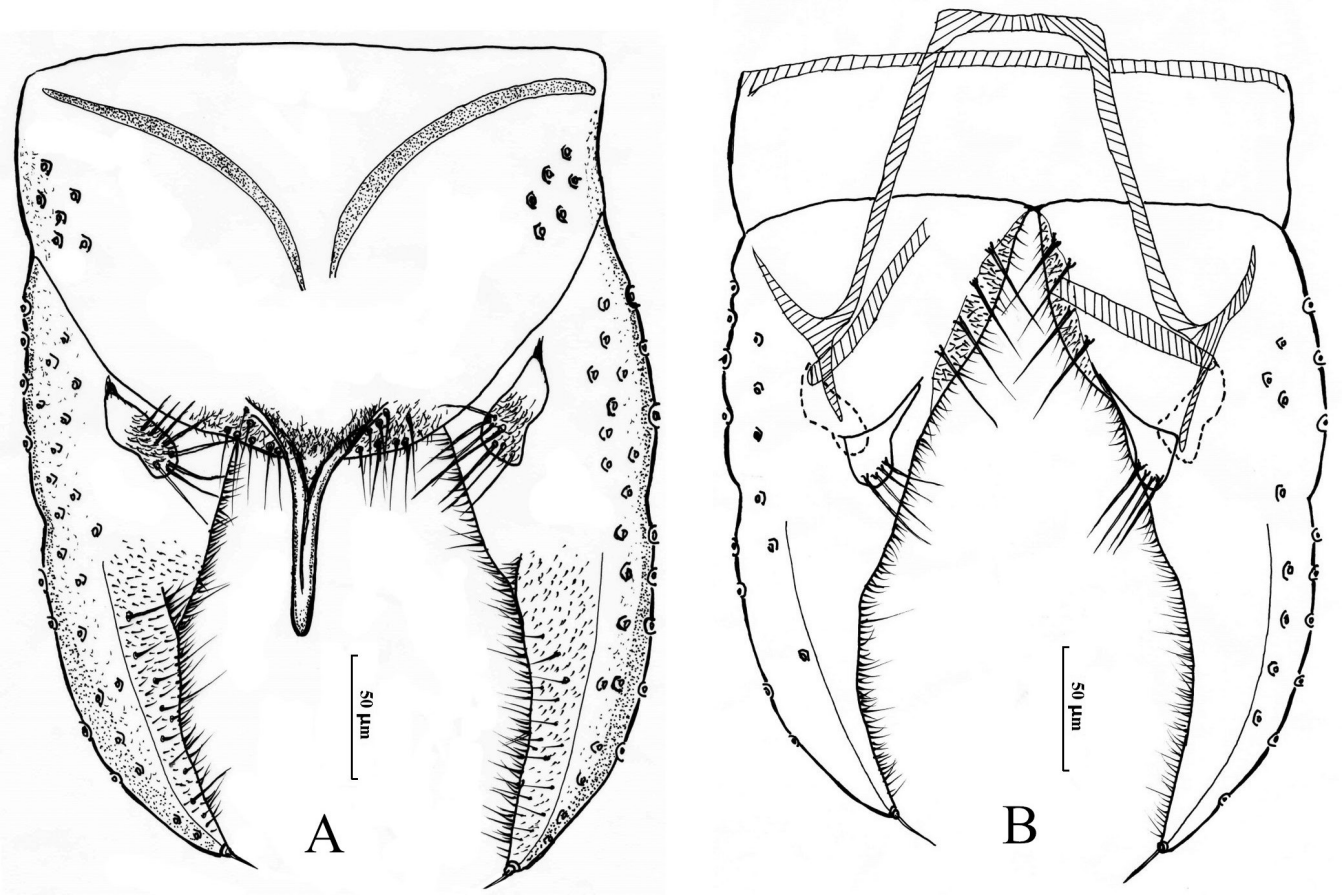
Male adult of *Cryptochironomus tonewabeus* (Sasa & Tanaka, 2002) comb. n., male. **A** hypopygium (dorsal view) **B** hypopygium (ventral view).

*Hypopygium* (Fig. 4A–B). Tergite IX shoulder-shaped at the posterior margin, bearing 16 setae. Laterosternite IX with six lateral setae. Anal point 77 μm long, wider at base, parallel-sided with rounded apex, lacking dorsal and lateral setae, microtrichia absent. Anal tergite bands “V”-shaped, not fused in the middle. Phallapodeme 90 μm long. Transverse sternapodeme 80 μm long. Superior volsella peanut-like, concave medially, bearing six strong setae. Inferior volsella thumb-like, with slender extension at the base, bearing four long setae at apex, free microtrichia. Gonocoxite 158 μm long, bearing eight strong setae along inner margin; Gonostylus 164 μm long, with seven short inner setae and one single seta. HR: 0.78; HV: 3. 25.

#### Distribution.

Japan.

#### Remarks.

Frontal tubercles cylindrical, superior volsella peanut-like, and concave medially combined with a thumb-like inferior volsella, bearing four long setae at apex, and free microtrichia followed the generic characters of Cranston et al. (1989). The “V” shaped anal tergite bands, not fused medially, are similar to *Cryptochironomus albofasciatus* (Staeger, 1839), but *C. tonewabeus* can be differentiated by the frontal tubercles, superior volsella and inferior volsella.

### Key to known male adults of *Cryptochironomus* from Japan

**Table:** 

1	Superior volsella spherical to bulbous	**2**
–	Superior volsella not as above	**7**
2	Junction of gonostylus and gonocoxite distinctly compacted	**3**
–	Junction of gonostylus and gonocoxite compacted slightly or not at all	**6**
3	Inferior volsella with microtrichia	***C. tamayoroi* Sasa & Ichimori, 1983**
–	Inferior volsella bare	**4**
4	Anal point tapering to the apex	**5**
–	Anal point nearly parallel-sided	***C. rostratus* (Kieffer, 1921)**
5	Tibia and tarsi of front legs dark brown, tibia with 3 subapical setae	***C. tamaichimori* Sasa & Kawai, 1987**
–	Tibia and tarsi of front legs light brown, tibia with 1 subapical seta	***C. tokaracedeus* Sasa & Suzuki, 1995**
6	Anal tergite bands “wide Y”-shaped	***C. misumaiprimus* (Sasa & Suzuki, 1998), comb. n.**
–	Anal tergite bands “H”-shaped	***C. tokaraefeus* (Sasa & Suzuki, 1995), comb. n.**
7	The posterior margin of tergite IX tapered	**8**
–	The posterior margin of tergite IX arced	**9**
8	Frontal tubercles low and broad, width is at least two times longer than height	***C. albofasciatus* (Staeger, 1839)**
–	Frontal tubercles tapering, width nearly equal to height	***C. hentonensis* Hasegawa & Sasa, 1987**
9	Anal point wide and short, gonostylus blunt and rounded apex	***C. javae* Kieffer, 1924**
–	Anal point slender and short, gonostylus tapering to the apex	**10**
10	Superior volsella spherical, slightly compacted at base	***C. jokaprimus* Sasa & Ogata, 1999**
–	Superior volsella peanut-like, distinctly compacted at distal 1/3	***C. tonewabeus* (Sasa & Tanaka, 2002), comb. n.**

## Supplementary Material

XML Treatment for Cryptochironomus albofasciatus

XML Treatment for Cryptochironomus hentonensis

XML Treatment for Cryptochironomus javae

XML Treatment for Cryptochironomus jokaprimus

XML Treatment for Cryptochironomus misumaiprimus

XML Treatment for Cryptochironomus rostratus

XML Treatment for Cryptochironomus tamaichimori

XML Treatment for Cryptochironomus tamayoroi

XML Treatment for Cryptochironomus tamayoroi

XML Treatment for Cryptochironomus tokaraefeus

XML Treatment for Cryptochironomus tonewabeus
